# 早期结外NK/T细胞淋巴瘤患者P-GemDOx方案疗效和安全性分析以及预后分层探索

**DOI:** 10.3760/cma.j.cn121090-20230726-00029

**Published:** 2024-02

**Authors:** 彤瑶 邢, 韦婷 王, 浩睿 申, 佳竹 吴, 华 尹, 悦 李, 莉 王, 金花 梁, 建勇 李, 卫 徐

**Affiliations:** 南京医科大学第一附属医院（江苏省人民医院）血液科，南京 210029 Department of Hematology, the First Affiliated Hospital of Nanjing Medical University (Jiangsu Province Hospital), Nanjing 210029, China

**Keywords:** 淋巴瘤, 结外NK-T细胞, 培门冬酶, 预后, 生存, Lymphoma, extranodal NK-T-Cell, Pegaspargase, Prognosis, Survival

## Abstract

**目的:**

探讨P-GemDOx方案一线治疗早期结外NK/T细胞淋巴瘤（ENKTL）患者的有效性、安全性以及相关预后因素。

**方法:**

回顾性分析2015年8月至2021年5月南京医科大学第一附属医院血液科收治的60例使用P-GemDOx方案治疗的早期初诊ENKTL患者的临床资料，使用*χ*^2^检验及Fisher确切概率法比较组间临床特征的差异，使用Log-rank检验比较组间生存差异，进行生存和预后因素分析。

**结果:**

60例患者在完成4～6个周期P-GemDOx方案治疗后，总缓解率（ORR）为88.3％，46例（76.7％）获得完全缓解（CR）。4年的无进展生存（PFS）率和总生存（OS）率分别为（66.3±7.1）％和（79.5±6.0）％。根据PINK/PINKE预后评分系统进行分组，各组间PFS和OS差异均无统计学意义。23.3％的患者24个月内出现疾病进展（POD<24），POD<24组（14例）和24个月内未出现疾病进展（POD≥24）组（46例）的OS期差异有统计学意义（*P*<0.001）。分析POD<24的危险因素，并在此基础上建立了预测POD<24的国际预后指数（POD24-IPI）预后分层模型（诊断时PET-CT检查存在病灶最大标准摄取值>12.8，1分；非单个鼻腔浸润，1分；4～6个周期治疗后疗效评估未达CR，1分；低危组， 0分；中危组，1分；高危组，2～3分），低危、中、高危组4年OS率分别为100％、（85.6±9.7）％、（65.0±10.2）％（*P*＝0.014）。此外，P-GemDOx化疗方案临床应用的总体耐受良好，主要不良反应是血液学毒性。

**结论:**

在早期ENKTL患者中，P-GemDOx方案是一个安全且有效的一线治疗方案；POD24-IPI是一个良好的风险分层模型。

## Abstract (en)

**Objective:**

To assess the efficacy, safety, and related prognostic factors associated with the P-GemDOx regimen as a first-line treatment for patients with early-stage extranodal natural killer （NK）/T cell lymphoma （ENKTL）.

**Methods:**

A retrospective analysis was performed on sixty early-stage ENKTL patients treated with the P-GemDOx regimen who were admitted to the First Affiliated Hospital of Nanjing Medical University between August 2015 and May 2021. The Chi-square test or Fisher's exact test was used to compare group differences, and the Log-rank test was used to compare the differences in survival. Survival outcomes and prognostic factors were examined.

**Results:**

After completing 4 to 6 cycles of P-GemDOx chemotherapy, the overall response rate （ORR） was 88.3％, with forty-six patients （76.7％） achieving complete response （CR）. The 4-year progression-free survival （PFS） and overall survival （OS） rates were （66.3±7.1）％ and （79.5±6.0）％, respectively. According to the PINK/PINK-E model, there was no significant difference in survival outcomes among risk groups. 23.3％ of patients experienced progression of disease within 24 months （POD<24）. OS estimates differed significantly （*P*<0.001） between the POD<24 group （*n*＝14） and the POD≥24 group （*n*＝46）. Analysis showed that SUVmax > 12.8 at diagnosis, non-single nasal cavity infiltration, and response less than CR after 4–6 cycles all had a significant association with POD24. We used these data as the basis for predicting POD<24 international prognostic index （POD24-IPI）. Patients were stratified into low-risk （no risk factors）, intermediate-risk （one risk factor）, or high risk （two or three risk factors）. These groups were associated with 4-year OS rate of 100％, （85.6±9.7）％, and （65.0±10.2）％, respectively （*P*＝0.014）. The P-GemDOx regimen was well tolerated, with hematological toxicity being the main side effect.

**Conclusion:**

This study demonstrated that the P-GemDOx regimen is effective and safe in the first-line treatment of early-stage ENKTL, and POD24-IPI is a promising prognostic model.

结外NK/T细胞淋巴瘤（ENKTL）是一种侵袭性恶性淋巴瘤，在亚洲和拉丁美洲更常见，且70％以上的ENKTL患者为早期[1]。目前以门冬酰胺酶为基础的化疗方案在治疗ENKTL患者中疗效肯定，如NCCN指南中推荐的改良SMILE、P-GEMOX和DDGP方案[2]，但ENKTL尚无标准化疗方案。因此，本中心针对早期ENKTL患者设计了以培门冬酶（门冬酰胺酶）为基础，联合GemDOx（吉西他滨、奥沙利铂、地塞米松）的治疗方案，命名为P-GemDOx方案，并自2015年起应用于临床实践中。

PINK/PINK-E是ENKTL的风险分层模型，但无法对早期ENKTL患者进行精准分层，大多数早期ENKTL患者的PINK/PINK-E风险分组为低中危[3]，约25％的早期ENKTL患者会在24个月内出现疾病进展（POD<24）[4]–[6]。既往研究表明，24个月内未出现疾病进展是弥漫性大B细胞淋巴瘤（DLBCL）、滤泡性淋巴瘤（FL）和外周T细胞淋巴瘤（PTCL）预后良好的标志[7]–[9]。24个月内未发生疾病进展的早期ENKTL患者5年总生存（OS）率为92.9％[4]。因此，我们推测POD<24是早期ENKTL患者预后不良的标志。本回顾性研究旨在探讨P-GemDOx方案一线治疗早期ENKTL患者有效性、安全性以及建立预测POD<24的国际预后指数（POD24-IPI）风险分层模型。

## 病例与方法

1. 病例：本研究纳入了2015年8月至2021年5月于南京医科大学第一附属医院血液科新诊断为早期ENKTL的60例患者。纳入标准如下：①根据世界卫生组织造血和淋巴组织肿瘤病理学分类诊断为ENKTL；②Ann Arbor分期为Ⅰ/Ⅱ期；③接受P-GemDOx方案进行诱导化疗至少3个周期，并进行有效评估；④人口统计学数据、基线临床特征和实验室检验资料完整。

2. 资料收集：从医院电子病历系统收集了基线人口统计学数据和临床特征，包括性别、年龄、美国东部肿瘤协作组（ECOG）评分、Ann Arbor分期、B症状（发热、盗汗、乏力或体重下降）、累及部位、PINK（年龄>60岁、Ⅲ～Ⅳ期、非鼻腔、远处淋巴结受累）和PINK-E（年龄>60岁、Ⅲ～Ⅳ期、非鼻腔、远处淋巴结受累、血浆EBV-DNA阳性）评分、实验室检查（LDH、全血EBV-DNA拷贝数）、影像学资料［治疗前^18^F-FDG PET/CT显像资料中病灶的最大标准摄取值（SUVmax）］。

3. 治疗方案：P-GemDOx方案：吉西他滨1 000 mg·m^−2^·d^−1^第1天、第5天，静脉滴注；奥沙利铂75 mg·m^−2^·d^−1^第1天，静脉滴注；地塞米松40 mg/d第1～4天，静脉滴注；培门冬酶2 500 U/m^2^第2天，肌肉注射；21 d为1个周期。所有患者计划接受4～6个周期的P-GemDOx方案治疗，在获得缓解后进行放射治疗（50～54 Gy/25～27 f）。

4. 疗效评估：于以下时间点进行疗效评估：3个周期治疗结束后、末次治疗（4～6个周期）结束后、治疗结束后的2年内每3个月1次。根据2014版Lugano疗效评定标准，分为完全缓解（CR）、部分缓解（PR）、疾病稳定（SD）和疾病进展（PD）。总缓解率（ORR）为CR率+PR率。

5. 安全性评价：根据《常见不良反应事件评价标准（CTCAE）》5.0版，对患者每个周期用药后发生的不良事件（AE）进行分级。

6. 随访：通过住院病历、门诊就诊记录和电话对所有患者进行随访。随访截止日期为2022年2月1日。随访结局事件包括无进展生存（PFS）期和OS期。OS期定义为从初始治疗至任何原因导致死亡或随访终止的时间。PFS期定义为从初始治疗至出现复发或进展的时间。疾病进展时间（POD）被定义为从初始治疗到疾病进展的时间间隔。POD<24指在24个月内发生疾病进展。POD≥24指24个月后发生疾病进展。

7. 统计学处理：患者的临床特征、疗效和AE等资料采用描述性统计分析，计数资料以例数（百分比）表示，计量资料以*M*（范围）或*x±s*表示。使用X-tile软件判断SUVmax最佳截断值。采用SPSS 26.0和GraphPad Prism 9软件进行统计学分析。组间比较采用*χ*^2^检验及Fisher确切概率法。采用Kaplan-Meier法计算PFS期和OS期，组间PFS期和OS期的比较采用Log-rank检验。*P*<0.05为差异有统计学意义。

## 结果

1. 临床特征：共60例初诊患者纳入分析，其中男43例，女17例；中位年龄52（21～73）岁，9例（15.0％）年龄>60岁；21例（35.0％）伴有B症状；9例（15.0％）ECOG评分≥2分；36例（60.0％）PINK-E评分≥1分，10例（16.7％）血清LDH>正常上限；31例（51.7％）全血EBV-DNA拷贝数≥500/ml。57例（95.0％）原发于上呼吸消化道（UAT），21例（35.0％）病灶局限于单个鼻腔。以SUVmax为12.8为临界值，24例（40.0％）患者SUVmax>12.8，36例患者（60.0％）SUVmax≤12.8。30例（50.0％）患者在3个周期治疗后达到CR，46例（76.7％）患者在6个周期治疗后达到CR，14例（23.3％）患者POD<24。根据是否在24个月内发生疾病进展，将60例患者分为POD<24组（14例）和POD≥24组（46例），比较两组间的临床特征，病灶局限于单个鼻腔（*P*＝0.0126）、4～6个周期治疗后达到CR（*P*＝0.0002）、SUVmax<12.8（*P*＝0.0341）与POD≥24密切相关。

2. 疗效评价： 60例患者共给予324个周期的P-GemDOx方案治疗，中位治疗6（4～6）个周期。ORR为88.3％（53/60），46例（76.7％）患者获得CR，7例（11.7％）患者获得PR，7例（11.7％）患者发生PD。42例患者完成了6个周期治疗，CR 34例（81.0％），PR 3例（7.1％），2例患者因疾病控制不佳更换方案，16例患者因个人原因停止治疗并在末次治疗结束后完成疗效评估［CR 12例（75.0％），PR 4例（25.0％）］。5例患者在3个周期治疗后达到PR，但在6个周期治疗后评估为PD。53例（CR 46例，PR 7例）获得缓解的患者中，40例患者在获得缓解后接受了放射治疗（50～54 Gy/25～27 f），13例患者因个人原因未进行放射治疗。缓解后放射治疗组有12.5％（5/40）患者发生PD，缓解后未放射治疗组有38.5％（5/13）患者发生PD。60例患者中位随访44（10～80）个月，中位PFS期和OS期均未达到。2年PFS率为（75.3±5.8）％，2年OS率为（87.3±4.5）％；4年PFS率为（66.3±7.1）％，4年OS率为（79.5±6.0）％。接受和未接受放射治疗的患者的PFS率（*P*＝0.035）和OS率（*P*<0.001）差异均有统计学意义（图1）。

**图1:**
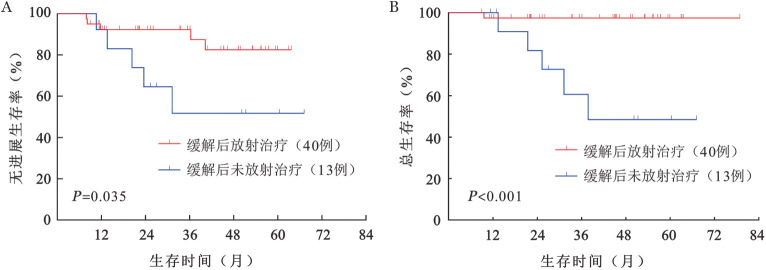
根据缓解后放射治疗和未放射治疗分组的早期结外NK/T细胞淋巴瘤患者无进展生存（A）和总生存（B）曲线

3. P-GemDOx方案安全性评估：P-GemDOx方案最常见的血液学不良反应为贫血（60例，100％）。3～4级中性粒细胞减少21例（35.0％）、3～4级贫血18例（30.0％）、3～4级白细胞减少14例（23.3％）、3～4级血小板减少11例（18.3％）。最常见的非血液学不良反应为纤维蛋白原降低（59例，98.3％）和低白蛋白血症（59例，98.3％）。3～4级纤维蛋白原降低12例（20.0％）、3～4级低白蛋白血症2例（3.3％）、3～4级胆红素增高1例（1.7％）、3～4级活化部分凝血活酶时间延长3例（5.0％）、3～4级丙转氨转氨酶增高3例（5.0％）、3～4级高血糖症4例（6.7％）、3～4级高甘油三酯血症2例（3.3％）、3～4级肌酸激酶升高1例（1.7％）、3～4级血清淀粉酶增高6例（10.0％）。胰腺炎3例（5.0％）。

4. POD24-IPI风险分层模型：51例（85.0％）患者PINK-E评分为0～1分（低危组），8例（13.3％）患者PINK-E评分为2分（低中危组），1例（1.7％）患者PINK-E评分为3分（高危组）。根据PINK、PINK-E预后评分系统进行分组，各组间PFS率、OS率差异均无统计学意义（图2）。POD<24是多种淋巴瘤的重要预后指标，包括ENKTL[4],[7]–[9]。分析OS显示，POD<24组和POD≥24组的OS差异有统计学意义（*P*<0.001），POD<24组4年OS率为（34.3±13.1）％，POD≥24组 4年OS率为（96.4±0.35）％（图3A）。比较POD<24组和POD≥24组患者的临床特征，结果表明SUVmax>12.8、4～6个周期治疗后疗效评估未达CR、非单个鼻腔浸润与POD<24相关，且PFS期、OS期较短，提示预后不良（图4）。在此基础上，我们建立了POD24-IPI（SUVmax>12.8，1分；4～6个周期治疗后疗效评估未达CR，1分；非单个鼻腔浸润，1分）。根据POD24-IPI将60例患者分为低危组（0分）、中危组（1分）和高危组（2～3分），各组间PFS（*P*<0.001）及OS（*P*＝0.014）差异具有统计学意义（图3B、C）。低危组4年PFS率为100％，4年OS率为100％；中危组4年PFS率为（73.7±11.8）％，4年OS率为（85.6±9.7）％；高危组4年PFS率为（42.1±11.2）％，4年OS率为（65.0±10.2）％。

**图2:**
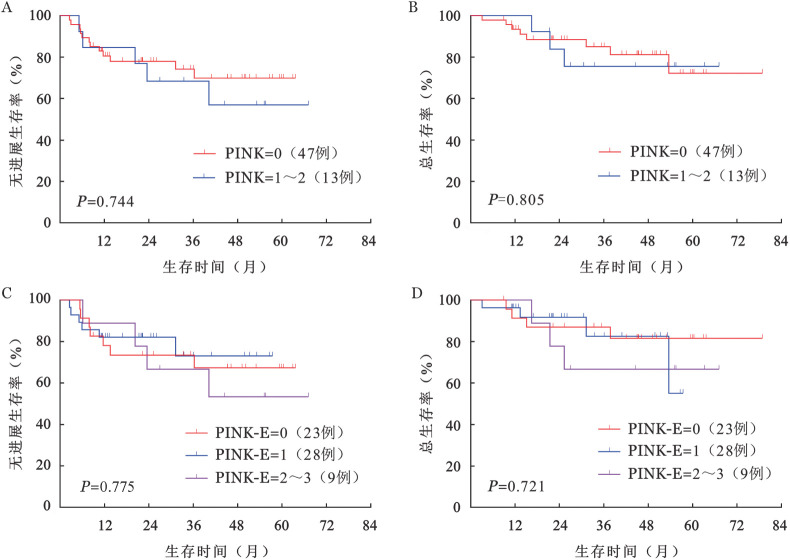
根据PINK及PINK-E分组的早期结外NK/T细胞淋巴瘤患者无进展生存和总生存曲线 A 根据PINK分组的无进展生存曲线；B 根据PINK分组的总生存曲线；C 根据PINK-E分组的无进展生存曲线；D 根据PINK-E分组的总生存曲线

**图3:**
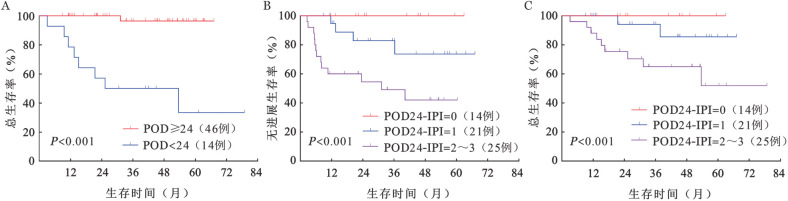
根据POD24-IPI分组的早期结外NK/T细胞淋巴瘤患者无进展生存和总生存曲线 A POD<24对总生存率的影响；B 根据POD24-IPI分组的无进展生存曲线；C 根据POD24-IPI分组的总生存曲线

**图4:**
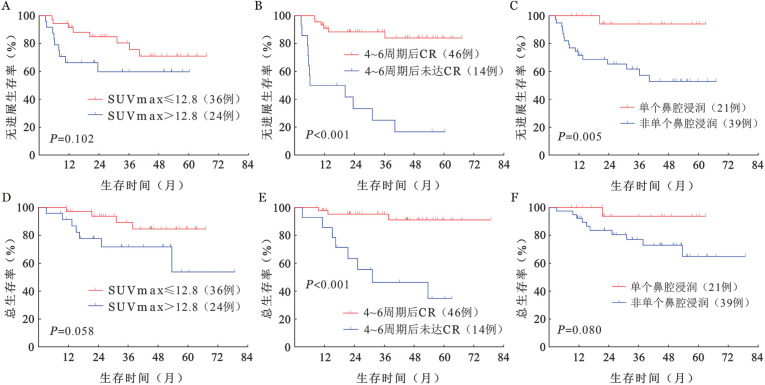
SUVmax、4~6周期治疗后完全缓解（CR）、单个鼻腔浸润对早期结外NK/T细胞淋巴瘤患者无进展生存及总生存的影响 A SUVmax对无进展生存率的影响；B SUVmax对总生存率的影响；C 4～6周期治疗后CR对无进展生存率的影响；D 4~6周期治疗后CR对总生存率的影响；E 单个鼻腔浸润对无进展生存率的影响；F 单个鼻腔浸润对总生存率的影响

## 讨论

早期ENKTL对放射治疗非常敏感，但它无法防止疾病全身性复发或进展[10]。目前，以培门冬酶为基础的治疗方案仍是ENKTL患者的首选。P-GemOx联合化疗方案在早期ENKTL患者中广泛使用。地塞米松是重要的抗淋巴瘤药物，也是治疗噬血细胞综合征的重要抗炎反应药物[11]，在与培门冬酶联合使用时，可以减少过敏反应和静脉血栓栓塞的风险[12]。在P-GemOx方案基础上，我们增加了大剂量地塞米松，命名为P-GemDOx方案。

本研究探讨了P-GemDOx方案作为早期ENKTL患者一线方案的有效性和安全性。60例初诊早期ENKTL患者经过4～6个周期P-GemDOx方案治疗后ORR为88.3％，76.7％患者获得CR，11.7％患者获得PR；2年PFS率为（75.3±5.8）％，2年OS率为（87.3±4.5）％；4年PFS率为（66.3±7.1）％，4年OS率为（79.5±6.0）％。血液学毒性是最常见的AE，非血液学毒性主要为纤维蛋白原降低和低白蛋白血症。NCCN指南推荐的方案有：联合化疗方案（SMILE、P-GEMOX、DDGP、AspaMetDex）、联合放化疗（DeVIC+RT、mSMILE+RT、P-GEMOX+RT）。早期应选择放疗和化疗综合治疗模式，SMILE、DDGP、AspaMetDex适用于晚期及复发难治的患者[2]。含培门冬酶的化疗方案是ENKTL最有效的全身化疗方案，SMILE方案在初治Ⅲ～Ⅳ期及复发难治的患者中疗效显著，但该方案骨髓抑制明显，治疗相关死亡率高达10％，且另一个明显的非血液学毒性是肾功能损害，mSMILE方案安全性较高，但仍有75％患者发生3～4级血液学毒性[13]。表1对不同化疗方案的有效性和安全性进行了总结。因此，P-GemDOx方案对早期ENKTL患者是安全且有效的，甚至可以与其他化疗方案相媲美。

**表1:** 含门冬酰胺酶方案一线治疗早期结外NK/T淋巴瘤的疗效

研究	例数	治疗方案	总缓解率（%）	完全缓解率（%）	无进展生存率（%）	总生存率（%）	3/4级中性粒细胞减少（%）
Jiang等[14]	26	LVP+RT	88.5	80.8	2年：80.6	2年：88.5	2.7
Wang等[15]	27	GELOX+RT	96.3	74.1	2年：86.0	5年：86.0	33.3
Kim等[16]	30	CCRT+VIDL	-	87.0	5年：60.0	5年：73.0	85.7
Guo等[17]	55	GOLD+RT	-	-	1年：87.0	1年：98.0	16.4
Bi等[18]	63	P-GEMOX/GELOX+RT	92.9	-	5年：55.9	5年：78.6	25.0
Dong等[10]	33	DICE-L+RT	100	90.9	5年：82.9	5年：89.2	75.8
Yoon等[19]	28	CCRT+MIDLE	-	57.1	3年：74.1	3年：81.5	91.3
Li等[1]	167	GELOXD/P-GEMOXD+RT	-	88.6	3年：72.8	3年：73.0	23.4
Wei等[6]	35	P-GEMOX+RT	94.3	80.0	2年：77.1	2年：82.9	37.1
Wei等[20]	36	SVILE(3 cycles)	91.7	38.9	2～3年：88.3	2～3年：88.8	84.7
	33	SVILE(3 cycles)+RT	91.7	83.4			
	33	P-GemOx(3 cycles)	97.0	39.4	2～3年：93.3	2～3年：97.0	64.8
	32	P-GemOx(3 cycles)+RT	97.0	97.0			
Liu等[5]	39	P-GDP	94.9	82.1	2年：80.8	2年：88.6	42.1
Yamaguchi等[21]	33	RT+DeVIC	78.0	75.0	5年：67.0	5年：73.0	93.0
Ghione等[15]	18	mSMILE+RT	94.0	89.0	31个月：92.0	31个月：100	64.0
Zhang等[3]	202	P-GEMOX+RT	94.1	63.9	3年：74.6	3年：85.2	24.8
本研究	60	P-GemDOx	88.3	71.7	4年：66.3±7.1	4年：79.5±6	35.0
					2年：75.3±5.8	2年:87.3±4.5	

注 GELOX：吉西他滨、奥沙利铂、左旋门冬酰胺酶；P-GEMOX/P-GemOx：培门冬酶、吉西他滨、奥沙利铂；GELOXD：吉西他滨、奥沙利铂、左旋门冬酰胺酶、地塞米松；P-GEMOXD/P-GOD：培门冬酶、吉西他滨、奥沙利铂、地塞米松；P-GDP：培门冬酶、吉西他滨、地塞米松、顺铂；RT：放射治疗； LVP：左旋门冬酰胺酶、长春新碱、泼尼松；CCRT：同步放化疗；VIDL：依托泊苷、异环磷酰胺、地塞米松、左旋门冬酰胺酶；GOLD：吉西他滨、奥沙利铂、左旋门冬酰胺酶、地塞米松；DICE-L：地塞米松、顺铂、依托泊苷、异环磷酰胺、左旋门冬酰胺酶；MIDLE：甲氨蝶呤、异环磷酰胺、地塞米松、左旋门冬酰胺酶、依托泊苷；SVILE：异环磷酰胺、培门冬酶、长春地辛、依托泊苷、地塞米松；DeVIC：地塞米松、依托泊苷、异环磷酰胺、卡铂；mSMILE：地塞米松、甲氨蝶呤、异环磷酰胺、培门冬酶、依托泊苷；3 cycles：3周期；-：暂无

PINK/PINK-E是以门冬酰胺酶化疗方案为基础，且经过大样本验证的ENKTL风险分层模型，广泛应用于临床实践中[2]，然而大多数早期ENKTL患者的PINK/PINK-E风险分组为低中危[3]。根据PINK/PINK-E对60例患者进行风险分层，47例（78％）患者PINK分组为低危组，51例（85％）患者PINK-E分组为低危组，各组间的PFS率和OS率差异无统计学意义，这与先前的研究结果一致[3]。PINK/PINK-E无法识别早期ENKTL患者中的高风险人群，然而超过70％的ENKTL患者诊断时为早期[1]，因此，我们迫切需要探索一种适用于早期ENKTL患者的风险分层模型。既往研究表明约25％早期ENKTL患者发生POD<24，POD<24被认为是早期进展，早期进展提示预后不良[4]–[6]。本研究结果显示，虽然88.3％的早期ENKTL患者经过治疗后可以获得缓解（PR或CR），仍有23.3％的患者发生POD<24。既往研究表明24个月内是否出现疾病进展是对DLBCL、PTCL和FL患者进行风险分层的重要标志[7]–[9]，也是评估ENKTL患者预后指标，初始治疗后出现早期进展的ENKTL患者即使疾病分期为早期，预后也极差[4]。在本研究中，我们观察到在使用同一化疗方案（P-GemDOx）作为一线治疗的早期ENKTL患者中，POD<24与较短的OS期相关。随后，我们确定了POD<24的危险因素，由此建立了POD24-IPI风险分层模型。根据POD24-IPI风险分层模型进行分组，各组间的PFS（*P*<0.001）、OS（*P*＝0.014）差异具有统计学意义。新的风险分层模型的应用可以促进精准治疗的实现。基于POD<24建立的风险分层模型不仅可以识别早期ENKTL患者中的高危患者，还能推动治疗方案的开发，为高危患者带来希望，有利于精准治疗的实现。当早期ENKTL患者在诊断时SUVmax>12.8且不是单一鼻腔浸润（POD24-IPI：2）时，可给予例如信迪利单抗或阿扎胞苷联用P-GemDOx方案、SC（信迪利单抗、西达本胺）方案等[22]。当早期ENKTL患者在4～6个周期P-GemDOx治疗后未达到CR时，应继续化疗而不是单纯放射化疗。

由于本研究是回顾性分析，且为单中心研究，样本量小，需前瞻性多中心研究且扩大样本量来验证P-GemDOx方案的有效性和安全性。POD24-IPI风险分层模型是在使用同一治疗方案的早期ENKTL患者中建立的，存在偏倚，在早期ENKTL患者应用的准确性需进一步验证。
